# To Clear Waste Pipes

**Published:** 1890-02

**Authors:** 


TO CLEAR WASTE PIPES.—A retired plumber thus gives a point for the gratuitous relief of householders: "Just before retiring at night pour into the clogged pipe enough liquid soda lye to fill the 'trap' or bent part of the pipe. Be sure that no water runs in it until the next morning. During the night the lye will convert all the offal into soft soap, and the first current of water in the morning will wash it away and clear the pipe clean as new."—*Medical Classics.*

